# Does ApoE promote phagocytosis of Amyloid‐beta?

**DOI:** 10.1002/alz70859_098129

**Published:** 2025-12-25

**Authors:** Xin Huang, Jennica Wang, Yoshiteru Kagawa, Abdel Ali Belaidi, Ben J Gu, Colin L Masters

**Affiliations:** ^1^ The University of Melbourne, Parkville, VIC Australia; ^2^ The Florey Institute of Neuroscience and Mental Health, Parkville, VIC Australia; ^3^ Innate Phagocytosis Laboratory, Jumar Bioincubator, Melbourne, VIC Australia; ^4^ The Florey Institute of Neuroscience and Mental Health, The University of Melbourne, Parkville, Melbourne, VIC Australia

## Abstract

**Background:**

The APOE ε4 allele is the strongest genetic risk factor for sporadic Alzheimer’s disease (AD), associated with earlier onset by ∼7.5 years compared to non‐ε4 carriers. Recent studies suggest ApoE4 promotes amyloid‐beta (Aβ) aggregation and lipid droplet formation, linking it to early pathogenesis. Interestingly, after onset, clinical progression appears similar between ε4 carriers and non‐carriers. This suggests ApoE4 triggers AD but may not significantly influence later progression. Whether ApoE modulates Aβ phagocytosis remains unresolved. This study investigates the effects of ApoE3 and ApoE4 on Aβ phagocytosis.

**Method:**

HEK293 cells stably expressing ApoE3 or ApoE4 were cultured in serum‐free media. ApoE proteins were isolated from conditioned media using Macrosep filtration (10 kDa Omega, Blue, PALL) and freeze‐drying (Martin Christ). Protein concentration was quantified by Western blot using anti‐pan ApoE and ApoE4‐specific antibodies. Phagocytosis assays were conducted using two complementary systems: 1) Real‐time flow cytometry: THP‐1 monocytes were exposed to Yellow‐Green (YG) fluorescent beads with or without ApoE3 or ApoE4 treatment. 2) Three‐colour fluorescence‐reporting system: BV2 microglia were treated with pHrodo‐red Aβ oligomers in the presence or absence of ApoE3 or ApoE4 for 24 or 48 hours. Surface binding of Aβ oligomers was evaluated using AF647‐labelled Aducanumab. LipidSpot 488 staining examined lipid droplet formation and phagocytic activity intensity.

**Result:**

ApoE proteins inhibited YG bead phagocytosis by THP‐1 cells dose‐dependently, with no significant differences between ApoE3 and ApoE4. Similarly, in BV2 cells, both ApoE isoforms reduced pHrodo‐red Aβ endocytosis and acidification. Both treatments also reduced lipid droplet formation, indicating lower phagocytic intensity. ApoE4 decreased Aβ oligomer surface binding compared to ApoE3.

**Conclusion:**

ApoE inhibits phagocytic activity in monocytes and microglia regardless of isoform, reducing lipid droplet formation and potentially limiting pro‐inflammatory M1 macrophage activation. The reduced Aβ surface binding by ApoE4 suggests diminished Aβ chaperoning, possibly contributing to Aβ aggregation initiation. These findings highlight mechanistic differences between ApoE isoforms in AD pathogenesis.

